# Motion aftereffects from viewing another’s gaze are restricted to the line of sight

**DOI:** 10.3389/fnhum.2026.1796537

**Published:** 2026-04-01

**Authors:** Christian Renet, Ronja Löfström, William Randall, Arvid Guterstam

**Affiliations:** 1 Department of Clinical Neuroscience, Karolinska Institute, Stockholm, Sweden; 2 Department of Psychology, Princeton University, Princeton, NJ, United States

**Keywords:** attention, gaze perception, implied motion, motion adaptation, social cognition

## Abstract

Recent work has shown that brief exposure to static images depicting an agent actively gazing at an object produces a motion aftereffect in the direction of the gaze. Such findings suggest that encoding of an implied agent-to-object motion takes place. Here, we adapted a previously used random dot motion paradigm, quantifying changes in perceptual decision thresholds for detecting left vs. right motion, to include probes in different spatial locations following the agent-and-object image. As with motion aftereffects from real motion, the aftereffects following exposure to the image were constrained to a limited portion of the visual field, specifically on and immediately above the line of sight of the agent in the image. The effect disappeared in control experiments featuring a blindfolded agent. These findings strongly support the notion that our brains encode others’ attentive gaze as an implied agent-to-object motion, and for the first time reveal the spatial extension of this internally generated motion signal.

## Introduction

Attention plays a central role in guiding behavior. As social animals, being able to track the attention of others is thus fundamental for constructing an accurate theory of mind and predicting others’ behavior (Baron-Cohen, 1997; Calder et al., 2002; Graziano and Kastner, 2011; Kobayashi and Kohshima, 1997). Across recent behavioral and brain imaging studies, evidence has accumulated that people construct a rich, implicit model of others’ attention that goes far beyond merely reconstructing others’ gaze vectors based on visual cues from their eyes (Guterstam et al., 2019, 2020, 2021; Guterstam and Graziano, 2020a, 2020b; Kelly et al., 2014; Pesquita et al., 2016; Ziman et al., 2023). Particularly, a behavioral motion aftereffect has been shown to follow brief exposure to static images of agents attending to objects (Guterstam and Graziano, 2020a; Renet et al., 2024). Motion aftereffects are generally assumed to reflect consequences of direction-specific neural fatigue on motion processing time and perceptual-decision making, and they are typically experimentally revealed as slower reaction times (RTs) and decreased accuracy for random dot motion test probes of the same directionality as the preceding motion stimulus, including implied motion (Anstis et al., 1998; Glasser et al., 2011; Kourtzi and Kanwisher, 2000; Krekelberg et al., 2003; Levinson and Sekuler, 1974; Winawer et al., 2010). The occurrence of a motion aftereffect after exposure to a static image of an agent actively gazing at an object therefore suggests the processing of an implied motion signal when viewing the image. It has also been shown that viewing low-level visual motion and gaze-images produces similar fMRI activity patterns in motion-related and social brain areas (Guterstam et al., 2020), and that people’s social cognitive decisions about the attention of others can be covertly manipulated by introducing subthreshold motion ‘beams’ between faces and objects (Guterstam and Graziano, 2020b). Together, these findings suggest that the visual motion system is involved in tracking the attention of other social agents in such a way that others’ attention is encoded as an implied motion streaming from a person toward the object of that person’s attention. We propose that the brain constructs this physically highly inaccurate model of the world to track others’ attention in a computationally easy manner, by which social mechanisms co-opt low-level sensory mechanisms to encode sources and targets of attention. This model remains subthreshold for visual experience and outside explicit awareness but nevertheless affect behavior in a meaningful manner.

While evidence for the existence of such a model is accumulating, its detailed properties are not yet described. Motion aftereffects resulting from simple real motion stimuli are typically constrained within the adapted region of the visual field (Knapen et al., 2009; Smith et al., 2000; von Grünau and Dubé, 1992; Wade et al., 1996). Thus, if the previously reported motion aftereffect following a static image of an agent actively gazing at an object indeed results from a model that connects the agent to the object with implied motion, the motion aftereffect should be spatially restricted to the line of sight of the agent. To test this hypothesis, we adapted a previously used paradigm (Renet et al., 2024) which was based on a random dot motion direction discrimination task. Rather than only showing the dot motion probe on the line of sight, as in the original study, the probe was here shown at five spatial locations, extending both vertically above and below the line of sight. We hypothesized that the motion aftereffect, manifested as a change in perceptual decision threshold for detecting left vs. right motion, should only occur when the probe was presented in the center position (i.e., on the line of sight of the agent), but not in the other four conditions in which the test probes were presented either above or below the center position. Furthermore, when the agent is blindfolded, we should not detect any motion aftereffect, regardless of the spatial location of the dot motion test probe.

## Methods

The present experiments were conducted exactly as described by Renet et al. (2024) with only two key changes: the different spatial locations for the dot motion probes, and control experiments featuring a blindfolded instead of an away-looking face.

### Participants

Participants were recruited through the online behavioral testing platform Prolific (Palan and Schitter, 2018), in such a way that each participant could only partake in one of the 10 experiments, ensuring naivety to the paradigm. All participants indicated normal or corrected-to-normal vision, English as first language, and no history of mental illness or cognitive impairment. All experimental methods and procedures were approved by the Princeton University Institutional Review Board and the Swedish Ethical Review Authority, and participants were required to indicate that they read and understood a consent form outlining their risks, benefits, monetary compensation, and confidentiality, and that they agreed to participate, before starting the 6–8 min session. A target sample size of 100 participants was chosen in line with the previous experiment (Renet et al., 2024). Due to the stringent exclusion criteria, which were identical to those in Renet et al. (2024) and implemented to maximize the quality of online behavioral testing data (which is notoriously noisy), final sample sizes for those included in the analysis were smaller and varied between experiments [experiment 1a, *n*_total_ = 98, *n*_included_ = 52, 17 females, mean age 24 (SD = 5); experiment 1b, *n*_total_ = 100, *n*_included_ = 53, 12 females, mean age 26 (SD = 8); experiment 1c, *n*_total_ = 94, *n*_included_ = 55, 18 females, mean age 26 (SD = 7); experiment 1d, *n*_total_ = 97, *n*_included_ = 59, 23 females, mean age 25 (SD = 5); experiment 1e, *n*_total_ = 97, *n*_included_ = 56, 21 females, mean age 26 (SD = 7); experiment 2a, *n*_total_ = 99, *n*_included_ = 42, 15 females, mean age 26 (SD = 4); experiment 2b, *n*_total_ = 98, *n*_included_ = 51, 21 females, mean age 31 (SD = 11); experiment 2c, *n*_total_ = 92, *n*_included_ = 51, 21 females, mean age 30 (SD = 9); experiment 2d, *n*_total_ = 104, *n*_included_ = 50, 18 females, mean age 31 (SD = 10); experiment 2e, *n*_total_ = 103, *n*_included_ = 53, 29 females, mean age 31 (SD = 9)].

### Exclusion criteria

Three predefined criteria (Renet et al., 2024) determined exclusions: (i) poor task performance (accuracy <80% in the “easiest” condition when 30% of the dots moved either left or right), (ii) poor curve fit (goodness of fit (*R*^2^) below 0.9, as poor curve fit disproportionately affects the estimation of the sigmoid central point), or (iii) failure to read the instructions carefully (determined by an instructional manipulation check (ICM) (Oppenheimer et al., 2009)). Exclusions based on poor task performance and failure to read instructions were applied before any data fitting took place. On average, across the 10 experiments, the exclusion rate was 47% (experiment 1a, n_excluded_ = 46 [47%]; experiment 1b, 47 [47%]; experiment 1c, 39 [41%]; experiment 1d, 38 [39%]; experiment 1e, 41 [42%]; experiment 2a, 57 [58%]; experiment 2b, 47 [48%]; experiment 2c, 41 [44%]; experiment 2d, 54 [52%]; experiment 2e, 50 [49%]). This high rate of exclusion was expected given the results of Renet et al. (2024), where the average exclusion rate was 45%. The most common reason for exclusion was poor performance on the dot motion task (the first of the three listed criteria), accounting for 75% of exclusions on average across the experiments. As discussed in Renet et al. (2024), the poor performance was likely due to the combination of relatively low motion coherence levels (max 30% compared to a fixed 40% level in Guterstam et al. (2020), where the exclusion rate was 19%) and lack of practice trials (subjects in this study did not undergo any practice sessions, because accuracy was our primary outcome, in contrast to Guterstam et al. (2020)).

### Apparatus

**Figure 1:**
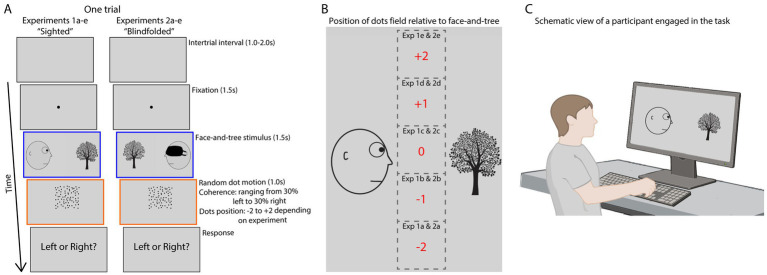
Depiction of one trial for each of the 10 experiments. **(A)** In experiments 1a–e, after a 1.0–2.0 s variable inter-trial interval, participants fixated a central spot for 1.5 s. Next a static, line-drawing image of a head looking at a tree was shown for 1.5 s with the head appearing left and looking right (shown here) on half of the trials and appearing right and looking left on the other half. After the image disappeared, a random dot motion stimulus was shown at one of five different vertical positions relative to the agent’s line of sight: 460 px below (exp 1a), 230 px below (exp 1b), in the line of sight (exp 1c), 230 px above (exp 1d), or 460 px above (exp 1e). A subset of the dots moved coherently either left or right. The dot coherence ranged from 30% of the dots moving left (and 70% moving in random directions) to 30% moving right, in 10% increments, yielding seven different types of motion. After the dots disappeared, subjects made a forced-choice judgement of the global direction of the moving-dots stimulus. As soon as the participant made a button press, the dots disappeared, and the next trial started. In experiments 2a–e, the sighted face in the static image was replaced by a blindfolded face, keeping all other experimental factors constant. **(B)** The positioning of the dots field with respect to the preceding face-and-tree image, in each of the 10 experiments. Note that while a sighted head is shown here, experiments 2a–e featured a blindfolded one. **(C)** A schematic view of a participant engaged in the task (Created in BioRender. Renet, C. (2026) https://BioRender.com/irfivq5).

Stimulus presentation and data collection were implemented on a website with custom software based on HTML, CSS, JavaScript [using the jsPsych javascript library (de Leeuw, 2015)], and PHP. Participants were redirected to this website after agreeing to participate and were required to complete the experiment in full screen mode. Viewing distance, screen size, and display resolution varied depending on the participants’ own setup (Figure 1C). The face-and-tree image took up 60% of the total screen width. Below, we report the stimulus dimensions using pixel [px] values for a screen with horizontal resolution of 1,050 px.

#### Experiment 1–experimental design and statistical analysis

The experiment started with the same written instructions as in the original study (Renet et al., 2024), and after the experimental session a survey followed that probed for knowledge of the hypothesis of the experiment. Figure 1A shows the structure of one trial of the behavioral paradigm. Inter-trial intervals had variable 1–2 s length, during which a neutral gray (R: 210, G: 210, B: 210) field covered the screen. Then, a black central fixation point (25 px diameter) appeared for 1.5 s, indicating the spatial location that participants were instructed to fixate on throughout the trial. After that, a static image of a face (228 px wide and 250 px high) gazing across the screen toward an arbitrary object, a tree (196 px wide and 250 px high), was shown for 1.5 s.

After the static image disappeared, a square-shaped random dot motion stimulus was presented for 1.0 s, centered horizontally and with vertical position varying across five different versions of experiment 1 (exp 1a-e), ranging from 460 px below to 460 px above the agent’s line of sight, as shown in Figure 1B. The random dot motion probe featured 400 black dots (2 px diameter) within a 230 × 230 px square. The speed of the dots was 2 px/frame, and lifetime was 12 frames (200 ms on a standard 60 Hz monitor). Seven different types of dot motion coherence levels were used: 30, 20%, or 10% moving either leftward or rightward (while the other 70, 80%, or 90% moved in random directions), plus one condition with 0% motion coherence (100% random). After the dots disappeared, participants were prompted about the direction of motion (“Left or Right?”). Trials were terminated upon a left or right arrow key button press, at which point the next trial began. The appearance of the face on the left and tree on the right, or the face on the right and tree on the left, were balanced and presented in random order, as was the case for the level of motion coherence. Overall, participants performed 70 trials in seven blocks of 10 trials each (10 trials per condition).

For the statistical analysis, the trial types were condensed into seven conditions of motion coherence: −30, −20%, −10%, 0%, +10%, +20%, and +30%. Trials with motion toward the location of the (preceding) tree were arbitrarily coded as having positive coherence, while motion away from the tree was coded as negative coherence. For each condition, we calculated the proportion of each participant’s responses that was spatially congruent with the direction away from the face and toward the tree (corresponding with the gaze of the face). We then fit the accuracy data to a sigmoidal function (Equation 1) (Noel et al., 2020) using the Curve Fitting Toolbox for MATLAB (MathWorks). The unbiased value of 0.5 was used as the starting point for the estimation of *x_c_* and *b* in Equation 1. We extracted the central point of the sigmoidal (*x_c_* in Equation 1) for each participant, indicating the level of dot coherence at which that participant was equally probable to indicate that the motion was moving toward the tree as compared to away from the tree.


$$f\;(x)=\frac{e^{\frac{x-x_{c}}{b}}}{1+e^{\frac{x-x_{c}}{b}}}$$ (1)

A central point significantly greater than 0 would indicate a motion aftereffect in the opposite direction of the agent’s gaze. As we hypothesized that the motion aftereffect resulting from visual input of another’s active gaze would be restricted to the visual space occupied by that other’s line of sight, we compared, at the group level, the mean central point to 0 using a one-sample two-tailed *t*-test. In experiment 1a, 1b, 1d, and 1e, the dot motion probe appeared below or above the line of sight of the agent in the preceding image, meaning we did not expect to find a significant motion adaptation effect in these experiments. We predicted that only in experiment 1c the mean central point would differ significantly from 0 (two-tailed one-sample *t*-tests). Bonferroni correction for multiple comparisons was applied for the five versions of experiment 1.

#### Experiment 2–experimental design and statistical analysis

The design, procedures, and statistical analysis of experiment 2 were identical to those of experiment 1, with one exception: experiments 2a-e featured a blindfolded face in the adapting stimulus (Figure 1A). The vertical position of the dot motion probe varied across experiments as in experiment 1a-e (Figure 1B). The experiments with a blindfolded face served as control, as any gaze-induced effect on motion judgments should be eliminated when the agent’s gaze is obstructed (Guterstam and Graziano, 2020a). Thus, we did not predict that the mean central point would differ significantly from 0 in experiment 2a-e (two-tailed one-sample *t*-tests). As in experiment 1, Bonferroni corrections were applied.

## Results

**Figure 2:**
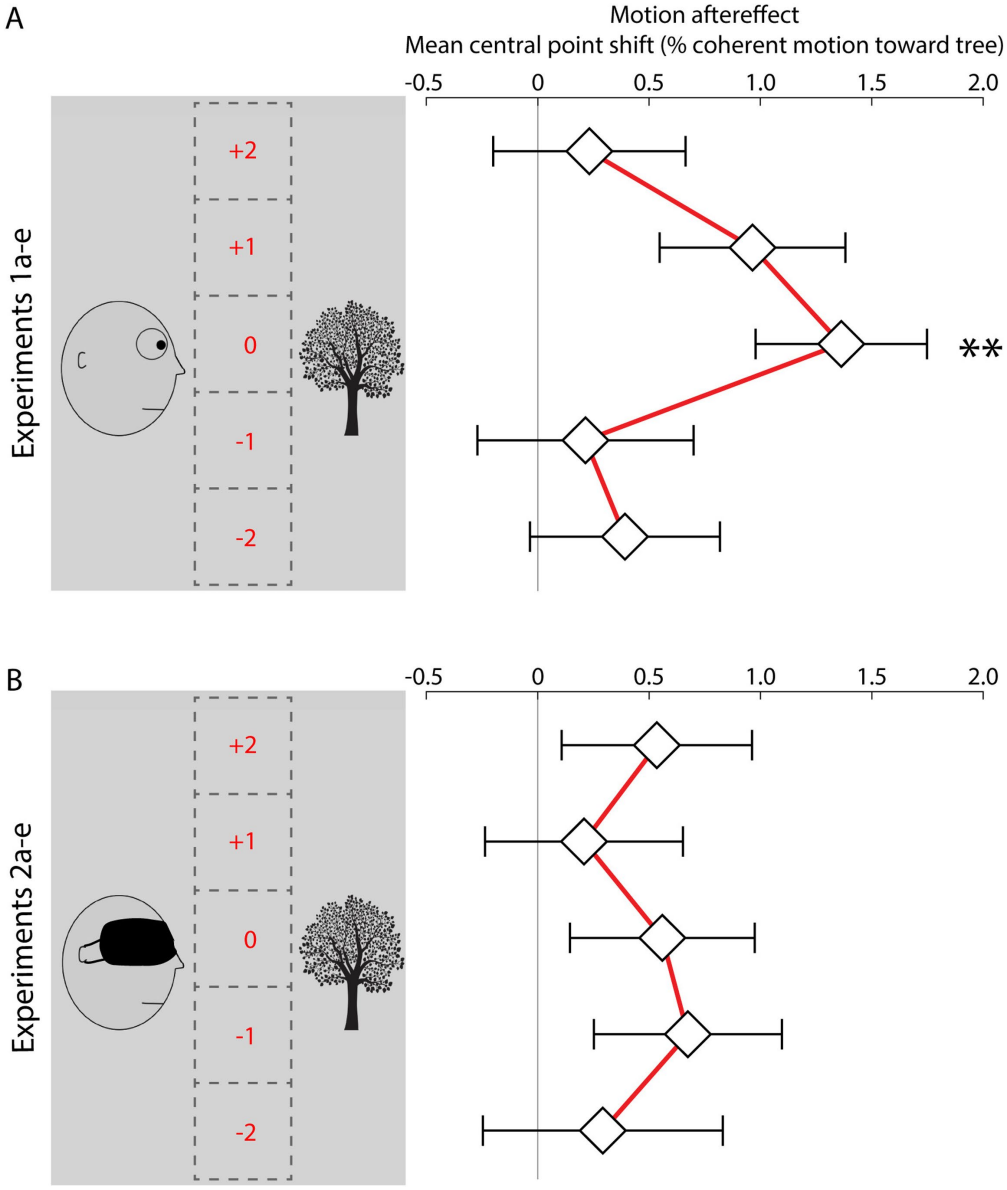
**(A)** Results for the experiments with a sighted face stimulus (1ae). The experiments differed only with respect to the vertical position of the dot-motion test probes (positions −2 to +2) in relation to the line of sight of the agent in the preceding image. Diamonds represent the mean central point of the sigmoid function for each experiment. Error bars represent the S.E.M. **(B)** Results for the control experiments with a blindfolded face (2ae). ^**^p < 0.01, Bonferroni-corrected.

Figures 2A,B summarize the results. Of the experiments with a sighted face stimulus, the mean central point of the sigmoidal function best describing subjects’ data across seven different dot motion coherence conditions was significantly greater than 0 only in experiment 1c (M = 1.36%, S.E.M. = 0.38%, t_54_ = 3.54, *p* = 0.004, Bonferroni-corrected, *d* = 0.48). In other words, immediately after the participants saw a face gazing in one direction, the amount of real motion needed to make them perceive that a test stimulus was randomly balanced between left and right movement was 1.36% (experiment 1c) coherence in the direction that the face had been gazing. This finding is consistent with a motion aftereffect to implied motion in the face-and-tree image streaming from the face to the tree. In experiments 1a, 1b, 1d, and 1e, the central point of the sigmoid function did not differ significantly from 0 (exp 1a: *M* = 0.39%, S.E.M. = 0.43%, *t*_51_ = 0.92, *p* = 1.0, *d* = 0.13; exp. 1b: *M* = 0.25%, S.E.M. = 0.49%, *t*_52_ = 0.44, *p* = 1.0, *d* = 0.06; exp. 1d: *M* = 0.96%, S.E.M. = 0.42%, *t*_58_ = 2.31, *p* = 0.121, *d* = 0.30; exp. 1e: *M* = 0.23%, S.E.M. = 0.43%, *t*_55_ = 0.54, *p* = 1, *d* = 0.07, all *p-*values Bonferroni-corrected; Figure 2A).

Across the control experiments with a blindfolded face stimulus, the central point of the sigmoid function was never significantly different from 0 (exp 2a: *M* = 0.29%, S.E.M. = 0.54%, *t*_41_ = 0.54, *p* = 1.0, *d* = 0.08; exp. 2b: *M* = 0.67%, S.E.M. = 0.42%, *t*_50_ = 1.60, *p* = 0.583, *d* = 0.22; exp. 2c: *M* = 0.55%, S.E.M. = 0.41%, *t*_50_ = 1.35, *p* = 0.919, *d* = 0.19; exp. 2d: *M* = 0.21%, S.E.M. = 0.44%, *t*_49_ = 0.47, *p* = 1.0, *d* = 0.07; exp. 2e: *M* = 0.53%, S.E.M. = 0.43%, *t*_52_ = 1.25, *p* = 1.0, *d* = 0.17, all *p-*values Bonferroni-corrected; Figure 2B).

In the post-experiment surveys, none of the participants indicated awareness of the true purpose of the experiment nor of any influence of the head-and-tree stimulus on their ability to respond to the dot motion stimulus, suggesting the reported motion adaptation effects occur at an implicit level. The data of all 10 experiments are available at https://doi.org/10.6084/m9.figshare.29664326.

## Discussion

Building on evidence that the brain treats the attentive gaze of others like an implicit motion (Guterstam et al., 2020; Guterstam and Graziano, 2020a; Renet et al., 2024), the present pattern of results strongly suggests that this implicit motion has defined spatial properties. We found that motion test probes were more likely to be judged as moving in the opposite, as compared to the same direction, as the gaze in the preceding image, compatible with a motion aftereffect to implied motion. The effect was only present when the depicted agent was actively gazing at the object, and only when the motion test probe was presented on the agent’s line of sight. These findings suggest that the motion-based internal representation of others’ attention, characterized in previous studies, is spatially constrained to the portion of empty space directly connecting social agents to the target of their attentive gaze.

Motion aftereffects from viewing real motion are restricted to the location in the visual field where such motion was shown (Knapen et al., 2009; Smith et al., 2000; von Grünau and Dubé, 1992; Wade et al., 1996). Our observation that the motion aftereffect from viewing static gaze stimuli is restricted to the part of the visual field between the eyes of the agent and the object adds strong support for the idea that the brain generates an implicit motion connecting the two. Combined with the fact that the effect disappears when the agent is blindfolded (and there is, consequently, no active gaze) or when the agent looks away [see experiment 2 in Renet et al. (2024)], these findings add additional support for the existence of an attention-motion model.

There exists much debate about what exactly is processed when people view the object-directed gaze of another person (Frischen et al., 2007). Considerable evidence suggests that the eye gaze of others serves as cue to redirect one’s own visual attention to the target of the other’s gaze (Emery, 2000; Frischen et al., 2007; McKay et al., 2021), even in infancy (Farroni et al., 2004). Others have found that eye gaze cues can lead participants to automatically take the visual perspective of the gazer (Samson et al., 2010). It has been argued that such perspective taking can be explained as a consequence of automatic attentional reorienting, although others have reported that automatic perspective taking is primarily driven by actions of the observed agent, not gaze (Furlanetto et al., 2013; Mazzarella et al., 2012). Moreover, it is unclear to what extent memory and attention processes that are not essentially social might support social processes (Happé et al., 2017; Heyes, 2014). In the context of this ongoing debate, our findings suggest that seeing another person’s object-directed gaze engages motion-related processes of the brain, as part of a much more complicated internal model of other’s attention than has previously been assumed. In this social attention model, eye gaze is a central cue that directs and spatially constrains a motion-based representation of others’ attention.

One limitation of our experiment is that, due to its online nature, experimental control was limited. Specifically, we could not obtain eye tracking data while participants were carrying out the task to confirm that fixation was maintained. It is reasonable to assume that participants maintained central gaze during the 1.5-s fixation and 1.5-s image phases of the trial. When the dot-motion probes were then presented in different spatial locations from the center, their appearance likely triggered reflexive saccades, as participants were not explicitly instructed to maintain central fixation during this part of the trial, and inhibiting such saccades typically requires explicit training and is especially difficult under competing task demands (Jamadar et al., 2015; Mitchell et al., 2002). However, for several reasons, this limitation does not fundamentally affect our conclusions. Firstly, reflexive saccades would have occurred directly upwards and downwards from the fixation point (i.e., completely orthogonal to the directions in which motion was shown). Such saccades would thus be unlikely to bias or interfere with participants’ left/right motion responses. Additionally, the effect that we report is specific to the eyes open condition. If the difference between the central location and the other locations in experiments 1a-e was driven by reflexive vertical saccades only, and not at all by whether the motion lined up with the gaze of the attending agent, the same pattern should have emerged in experiments 2a-e (eyes covered condition). Lastly, and most crucially, we specifically tested for a difference between congruent and incongruent trials in the same spatial location, meaning that any overall effect of reflexive saccades on dot-motion responses should affect the congruent and incongruent trials equally, leaving our key congruent-vs-incongruent outcome unaffected.

Assuming participants indeed made reflexive vertical saccades to the locations of the non-central probes, our results would suggest that adaptation to implied motion operates in a spatiotopic reference frame. It has been shown that the classic motion adaptation effect, following from real motion, is retinotopic (i.e., adaptation transfers with saccades so that the effect occurs at the retinal location of the adapting stimulus, not the fixed location in the outside world) (Knapen et al., 2009). However, to the best of the authors’ knowledge, it remains unclear whether this also applies to adaptation to implied motion, and, more specifically, to adaptation to implied motion associated with social gaze stimuli. Implied motion (including the internally generated motion signal associated with social gaze stimuli) has been shown to lead to activation in the medial temporal/medial superior temporal cortex (MT/MST) (Guterstam et al., 2020; Kourtzi and Kanwisher, 2000; Osaka et al., 2010) which, along with other visual areas, has been suggested to encode motion signals in a spatiotopic fashion (d’Avossa et al., 2007; Zimmermann et al., 2016). Furthermore, it has been shown that variations of the classic motion aftereffect occurring at higher levels of processing are spatially selective in external rather than retinal coordinates (Turi and Burr, 2012). Theoretically, it seems likely that high-level representations, such as a face gazing attentively at an object, are represented (at least partially) in the spatiotopic reference frames necessary for our perception of a stable external world (Burr and Morrone, 2011). Our results are thus consistent with this idea, since adaptation did not transfer with reflexive saccades to the locations of the probes, as it should have done if adaptation to implied motion occurred in a retinotopic reference frame. Future replications of the present experiment featuring eye tracking will be necessary to generate more conclusive evidence on this aspect of adaptation to implied motion.

In conclusion, our findings directly replicate the main result of Renet et al. (2024) in an independent cohort, with new control conditions, strongly supporting the notion viewing others attentively gazing at object produces genuine motion aftereffect. Crucially, they define, for the first time, the spatial extension of an implicit, motion-based model for processing others’ attention.

## Data Availability

The datasets presented in this study can be found in online repositories. The names of the repository/repositories and accession number(s) can be found in the article/supplementary material.
